# Gardnerella vaginalis Urinary Tract Infection in a Term Neonate With Enteroviral Meningitis

**DOI:** 10.7759/cureus.104561

**Published:** 2026-03-02

**Authors:** Maximillian Y Lee, Alvaro G Valdivia-Trujillo, Pablo J Sanchez

**Affiliations:** 1 Pediatrics, The Ohio State University College of Medicine, Columbus, USA; 2 Infectious Disease, Nationwide Children’s Hospital, Columbus, USA

**Keywords:** enterovirus, fever, gardnerella vaginalis, meningitis, neonate, sepsis

## Abstract

Vertical transmission of *Gardnerella vaginalis* is rare but may be associated with neonatal morbidity and mortality. We present a febrile neonate who was diagnosed with enteroviral meningitis and had a concomitant urinary tract infection (UTI) with *G. vaginalis*. Our case highlights the need to investigate for both bacterial and viral pathogens in neonates who present with possible serious infection.

## Introduction

*Gardnerella vaginalis *is a common inhabitant of the female urogenital tract, yet vertical transmission is rare. We report a full-term febrile neonate who was hospitalized with enteroviral meningitis and had a concomitant urinary tract infection (UTI) due to *G. vaginalis*. In addition, we reviewed the published English literature on neonatal infections caused by *G. vaginalis* [1-10]. Our objective is to alert pediatric health care professionals to this infrequent infection while highlighting the need for complete viral and bacterial evaluation of febrile neonates.

## Case presentation

A 3,743-gram female neonate was born vaginally at 39 weeks of gestation following induction of labor to a 31-year-old, gravida 5, para 4 Caucasian mother. She reported no recent illness or fever. Testing for hepatitis B surface antigen, hepatitis C antibody, *Neisseria gonorrhoeae*, *Chlamydia trachomatis*, group B *Streptococcus*, and HIV was negative. The appearance, pulse, grimace, activity, and respiration (APGAR) scores were 8 and 9 at one and five minutes, respectively. The infant’s growth parameters were appropriate for gestational age, and the physical examination was normal. The infant was discharged at 24 hours, receiving expressed maternal milk. 

At five days of age, the infant was seen by the pediatrician; the infant's weight had decreased 7.8% from birth but was otherwise well. At 12 days old, the infant was again seen by a pediatrician for weight assessment and found to have a temperature of 100.7°F. The infant appeared otherwise well without irritability, somnolence, or poor feeding, but the father and older brother had gastroenteritis. The infant was referred to the emergency department for sepsis evaluation, where she had a temperature of 98.4°F. She was nontoxic with no respiratory distress or rash but had slight yellow drainage from both medial canthi and a soft systolic murmur. In the emergency department, the patient underwent a full sepsis evaluation that included blood culture, catheterized urine for microscopic analysis and culture, lumbar puncture for CSF culture and PCR (Biofire^TM^, Salt Lake City, UT, USA) testing, nasopharyngeal swab for respiratory viral PCR (Biofire^TM^), and mucosal swabs and blood PCR for herpes simplex virus and enterovirus/parechovirus. 

On physical examination, the infant was afebrile and hemodynamically stable with no respiratory distress but had a mild maculopapular rash on extremities and back, a grade II systolic murmur, mild hepatosplenomegaly, and mild focal hypotonia with head lag and shoulder laxity. Results of the evaluation were significant for CSF analysis that showed 412 nucleated cells/mm³ (70% monocytes, 18% lymphocytes, and 7% polymorphonucleocytes), 89 red blood cells/mm³, protein of 108 mg/dL, and glucose of 37 mg/dL. Empiric therapy with intravenous cefotaxime, gentamicin, ampicillin, and acyclovir was initiated, and the infant was admitted to the Infectious Diseases service at Nationwide Children’s Hospital (Columbus, OH, USA). 

The CSF enteroviral PCR test was positive, but the bacterial culture was sterile. Mucosal swabs were positive for enterovirus, but blood enterovirus PCR was negative. The respiratory infection array also yielded rhinovirus/enterovirus. The complete blood cell and platelet count was normal, while the serum alanine aminotransferase (ALT) and aspartate aminotransferase (AST) concentrations were elevated at 94 U/L and 67 U/L, respectively. Total bilirubin concentration was 0.2 mg/dL, and the direct fraction was <0.1 mg/dL. Serum ferritin concentration was elevated at 1,445 ng/mL. Urinalysis showed pyuria with 26 WBCs/high power field (HPF), a large amount of leukocyte esterase, and occasional bacteria, with the urine culture subsequently positive for >100,000 colony-forming units/mL of *G. vaginalis*. A repeat blood culture was sterile; therefore, after 19 hours of incubation, the blood culture yielded *Staphylococcus epidermidis*, which was assessed as a contaminant. 

Additional evaluation included a normal echocardiogram as well as normal renal and cranial ultrasounds. Antimicrobial therapy was changed to clindamycin (10 mg/kg/dose q8 hours) for the *G. vaginalis *UTI, and the infant was discharged home to complete a seven-day course of oral clindamycin. Results of susceptibility testing were not known until after discharge; the organism was susceptible to penicillin, ampicillin, clindamycin, vancomycin, and meropenem. Two weeks after discharge, the infant was seen in follow-up and was thriving, weighing 3,875 grams and with normal serum transaminase concentrations (ALT 32 U/L; AST 44 U/L). 

## Discussion

*Gardnerella vaginalis*, or *Haemophilus vaginalis* as it was named when discovered in 1955, is a gram-variable, rod-shaped, facultative anaerobic bacteria that is associated with bacterial vaginosis but only rarely causes neonatal disease [1-10]. Our patient was a 12-day-old infant who presented with a low-grade fever and was diagnosed with enteroviral hepatitis and meningitis, only to have a concomitant UTI due to *G. vaginalis*. Viral and bacterial co-infection is infrequent, although knowledge of its occurrence is important for therapeutic intervention and supportive therapy. 

Vertical transmission of *G. vaginalis* occurs from natal exposure during vaginal delivery or by an ascending route following rupture of fetal membranes with or without chorioamnionitis. In addition, maternal bacteremia with *G. vaginalis* has been reported, so the possibility of transplacental transmission exists [11]. Our patient’s mother had not been diagnosed with bacterial vaginosis nor had she had a fever or vaginal discharge during pregnancy or postpartum. As our patient presented at 12 days of age and had a negative blood culture for *G. vaginalis*, she likely was colonized at delivery and subsequently developed a lower UTI by an ascending route. 

Antimicrobial therapy for infections due to *G. vaginalis *has focused mainly on adults with genital tract infections. Metronidazole and clindamycin are recommended for the treatment of bacterial vaginosis because of its polymicrobial nature, but *G. vaginalis* is often susceptible to penicillin, ampicillin, erythromycin, clindamycin, and vancomycin while resistant to amikacin and sulfamethoxazole. Similarly, our patient’s isolate was susceptible to penicillin, ampicillin, clindamycin, vancomycin, and meropenem. The infant received initial treatment with intravenous ampicillin followed by oral clindamycin since susceptibility testing was not available at the time of discharge. Previously reported cases of neonatal *G. vaginalis* infections received treatment with either metronidazole or a beta-lactam agent (Table 1) [1-10]. Overall mortality among the 18 reported cases was 18%, with the organism isolated from the blood of two preterm stillborn infants [8,10].

**Table 1:** Reported neonatal cases of culture-confirmed G. vaginalis infections *Diamniotic, dichorionic twins; APGAR: Appearance, pulse, grimace, activity, and respiration

Authors and year of publication		Gestational age	Birth weight	Sex	Delivery type; Birth complications	Age at diagnosis; Positive culture site	Treatment	Outcome
Current case	39 weeks		3743 g	Female	Vaginal delivery; none	12 days; Urine	Clindamycin (oral)	Resolved
Hamod et al., 2017 [1]*	33 weeks		1890 g	Female	Cesarean section; chorioamnionitis, preterm labor at 29 weeks, respiratory distress	Four days; Blood, gastric (swab), ear (swab), anus (swab)	Metronidazole (intravenous)	Resolved
Amaya et al., 2002 [2]	23 weeks		560 g	Female	Vaginal delivery; respiratory distress	Zero days; blood and tracheal aspirate	Metronidazole (intravenous)	Resolved
Zabé-Desanges et al., 2000 [3]	33 weeks		1540 g	Female	Cesarean section; premature rupture of membranes at 27 weeks; clinical sepsis, respiratory distress	Zero days; pharyngeal culture; gastric (swab), ear (swab), placenta (swab)	Cefotaxime, amoxicillin, and netilmicin	Resolved
Berardi-Grassias et al., 1988 [4]	37 weeks		2980 g	Female	Vaginal delivery; none	Five days; CSF	Ampicillin, cefotaxime, netilmicin	Resolved
Furman et al., 1988 [5]	41 weeks		3560 g	Female	Vaginal delivery; respiratory distress	Zero days; tracheal aspirate	Ampicillin, gentamicin	Resolved
Chowdhury et al., 1985 [6]	Full-term		3000 g	Male	Premature rupture of membranes	48 hours; conjunctivae	Topical chloramphenicol	Resolved
Leighton et al., 1982 [7]	38 weeks		3200 g	Male	Forceps-related cheek abrasions	10 hours; cheek abscess	Cloxacillin	Resolved
Venkataramani et al., 1976 [8]	Preterm		1600 g	-	Vaginal delivery (in ambulance)	Blood	Penicillin and kanamycin	Died (13 hours of age)
	Preterm		1200 g	-	Vaginal delivery; chorioamnionitis	Blood	Penicillin and kanamycin	Died (six hours of age)
	Preterm		2100 g	-	Premature and prolonged (48 hours) rupture of membranes	Zero days; blood	Penicillin and kanamycin	Resolved
	Term		2680 g	-	None	Zero days; blood	None	Resolved
Carney et al., 1973 [9]	Term		2820 g	Female	Cesarean section; chorioamnionitis; prolonged rupture of membranes (49 hours); meconium staining	Zero days; blood	Penicillin and kanamycin	Resolved
Platt et al., 1971 [10]	42 weeks		2285 g	Female	Vaginal delivery, APGAR 2 (5 minutes)	Zero days; blood	None	Died
	37 weeks		2800 g	Female	Vaginal delivery; forceps-assisted delivery	Four days; scalp abscess	Ampicillin and kanamycin, topical phisohex	Resolved
	-		-	-	-	Umbilical cord blood	-	Stillborn
	-		-	-	-	Umbilical cord blood	-	Stillborn

## Conclusions

We report a rare neonatal case of *G. vaginalis* UTI that was associated with enteroviral meningitis. Our case highlights the need to investigate for both bacterial and viral pathogens in neonates who present with possible serious infection. Early recognition and comprehensive diagnostic evaluation are essential to ensure timely, targeted management in neonates.
